# Supplementary material for: HMGB1-Mediated Cell Death—A Crucial Element in Post-Hepatectomy Liver Failure
Source: Int J Mol Sci. 2024 Jun 28;25(13):7150. doi: 10.3390/ijms25137150 (PMC11241647; doi:10.3390/ijms25137150)
Supplement: Supplementary file 1 [file ijms-25-07150-s001.zip › ijms-3055329-supplementary.pdf]

HMGB1-Mediated Cell Death - a Crucial Element in Post-Hepatectomy Liver Failure

Laura Brunnthaler[1], Thomas G Hammond, David Pereyra[2], Jonas Santol[1,3], Joel Probst[2], Valerie Laferl[2], Ulrike Resch[1], Monika Aiad[2], Anna Sofie Janoschek[2], Thomas Gruenberger[5], Patrick Starlinger[2,6*], Alice Assinger[1*]

[1]Department of Vascular Biology and Thrombosis Research, Centre of Physiology and Pharmacology, Medical University of Vienna, Vienna, Austria

[2]Department of General Surgery, Division of Visceral Surgery, Medical University of Vienna, General Hospital, Vienna, Austria

[3]Center for Biomedical Research, Medical University of Vienna, Austria

[4]Institute of Bioinformatics, Biocenter, Medical University of Innsbruck, Innsbruck, Austria

[5]Department of Surgery, HPB Center, Viennese Health Network, Clinic Favoriten and Sigmund Freud Private University, Vienna, Austria

[6]Department of Surgery, Division of Hepatobiliary and Pancreatic Surgery, Mayo Clinic, Rochester, MN, USA

*shared correspondence

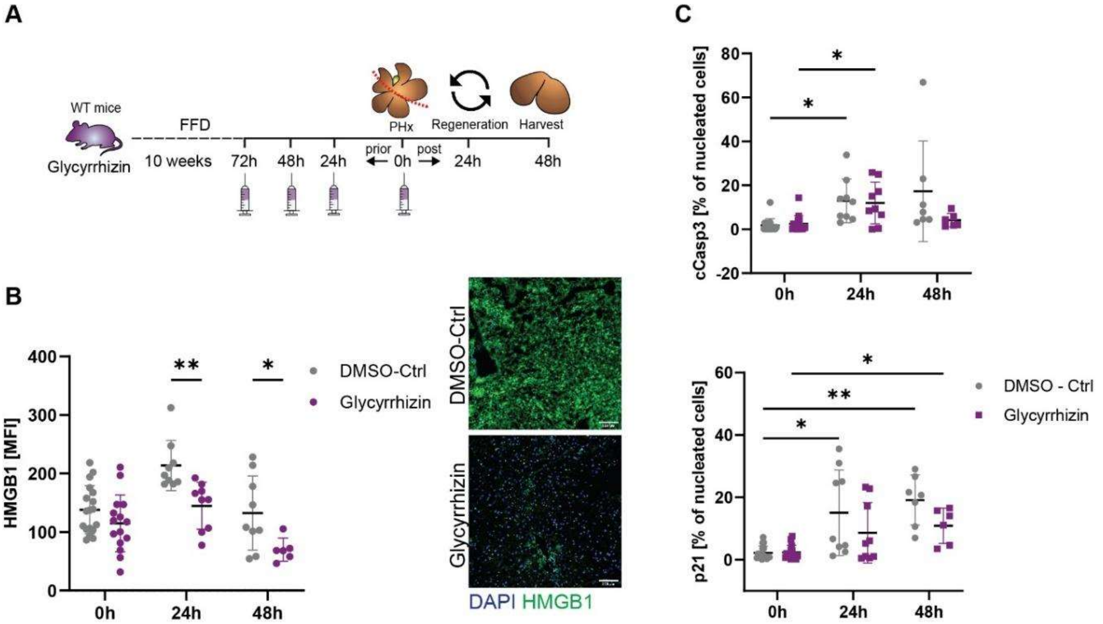

Figure S1: (A) Experimental scheme. 15-week-old WT mice on a fast food diet, treated with dimethyl sulfoxide-control (DMSO-Ctrl) or glycyrrhizin before being subjected to 70% PHx. **(B)** High mobility group box protein-1 (HMGB1) staining and quantification in mouse hepatic sections before and after PHx. (Two-way ANOVA: *p<0.05, **p<0.01). **(C)** Cleaved caspase 3 (cCasp3) and p21 staining and quantification in mouse hepatic sections before and after PHx. (Two-way ANOVA: *p<0.05).

Supplemental Methods

Quantification of Serum HMGB1

Total serum HMGB1 concentration was determined using the Shino-Test enzyme-linked immunosorbent assay (ELISA) (ST51011, IBL International, Tecan, Mannedorf, Switzerland) using manufacturer's instructions.

Samples were assayed using the normal test procedure exhibiting a range of quantification between 2.5 to 80 ng/ml. Samples quantified below this value were subsequently re-assessed using a high-sensitivity protocol with a range of quantification between 0.1 to 10 ng/ml. Reagents were allowed to warm to room temperature and serum samples were thawed on ice. Once thawed, samples were centrifuged at 2,000g for 1 minute. Lyophilised calibrator stock and positive controls were reconstituted using dilutent buffer volumes indicated on respective labels and left on ice for 10 mins. Calibrator standards were prepared following serial dilution of the reconstituted calibration stock solution. In the normal test procedure 100 µl of dilutent buffer was added to each well before addition of 10 µl of the calibrator standard, positive control or serum sample. In the high-sensitivity protocol 50 µl dilutent buffer was added to each well before addition of 50µl of the calibrator standard, positive control or serum sample. In both assays the plate was then sealed in adhesive foil, gently shaken and then incubated at 37°C for 24 hrs. After incubation, the lyophilised enzyme conjugate was reconstituted using the enzyme conjugate dilutent and left to stand for 10 minutes. Meanwhile the wash buffer was diluted through addition of 400 ml distilled water to 100 ml wash buffer. The plate was subsequently washed five times through addition of 400 µl wash buffer per well. The plate was then dried manually, and the lyophilised enzyme conjugate was reconstituted in 12 ml of the enzyme conjugate dilutent. 100 µl of the reconstituted enzyme conjugate was added per well, with the plate sealed and incubated at 25°C for 2 hours. After this the plate was washed a further five times through addition of 400 µl of the diluted wash buffer per well, before again manually drying the plate. Equal volumes of colour reagent A and B were then mixed, before addition of 100 µl of this mixture per well. The plate was then incubated for 30 minutes at room temperature in the dark. After this, 100 µl of the stop solution was added to each well with the plate gently shaken, and after 5 minutes the plate was read at 450 nm using a Varioscan Flash machine (Thermo Scientific, Basel, Switzerland). All samples were assayed in duplicate.

Quantification of Serum Keratin-18 Isoforms

Caspase-cleaved keratin-18 (ccK18) and total keratin-18 (total-K18) were determined using the M30 Apoptosense® and M65® Classic ELISA kits respectively (both Peviva®, TecoMedical, Sissach, Switzerland) according to the manufacturer's instructions.

Reagents were allowed to warm to room temperature and serum samples were thawed on ice. Once thawed, samples were centrifuged at 2,000g for 1 minute. For the M30 ELISA kit the wash buffer was prepared through dissolving one wash tablet in 500 ml deionised water. For the M65 ELISA kit the wash solution was diluted 10-fold in deionised water before use. The M30 HRP conjugate was diluted in 9.2 ml M30 Conjugate dilution buffer. The M65 HRP conjugate was diluted in 9.2 ml of M65 Conjugate dilution buffer. The quantification range of the M30 assay was 40 – 1000 U/L, and that of the M65 assay was 11 – 2000 U/L. Samples found to exceed the highest quantifiable range of the kits were diluted in blank calibration standard (0 U/µl standard) and re-assayed. For both kits 25µl of calibration standard, assay control standard or serum sample were applied to each well followed by 75 µl of corresponding conjugate solution. The plate was then sealed and incubated (4 hours for the M30 kit, 2 hours for the M65 kit) at room temperature on a plate shaker at 600 rpm. After this the wells were washed five times using 250 µl/well wash solution, before they were dried manually. After this 200 µl TMB substrate solution was added to each well and the plate was incubated at room temperature for 20 minutes in the dark. Subsequently 50 µl substrate solution was added to each well, and the plate was shaken for 10 seconds to ensure thorough mixing. After 5 minutes the plate was read at 450 nm using a Varioscan Flash machine (Thermo Scientific, Basel, Switzerland). All samples were assayed in duplicate.
